# Update on the Laboratory Diagnosis of Lupus Anticoagulant: Current Challenges and Clinical Involvement

**DOI:** 10.3390/jcm14082791

**Published:** 2025-04-18

**Authors:** Ana Marco-Rico

**Affiliations:** 1 Thrombosis and Hemostasis Department, Hematology Service, Hospital General Universitario Dr. Balmis, 03010 Alicante, Spain; marco_anaric@gva.es; 2 Instituto de Investigación Biomédica de Alicante (ISABIAL), 03010 Alicante, Spain; 3 Clinical Medicine Department, Universidad Miguel Hernández, 03550 Alicante, Spain

**Keywords:** lupus anticoagulant, antiphospholipid syndrome, preanalytical phase, dilute Russell’s viper venom test, silica clotting time, ratio, anticoagulation

## Abstract

Lupus anticoagulant (LAC) is a heterogeneous mix of autoimmune antibodies that prolongs phospholipid-dependent clotting assays. Its diagnosis can be a real challenge in the hemostasis laboratory. In this review, the author describes the main pitfalls affecting the preanalytical phase and how to proceed to reduce interferences. Because of the heterogeneity of these autoantibodies, two assays with different mechanism of action should be performed to detect the majority of LACs. The dilute Russell’s viper venom test and the use of a reagent very sensitive to LAC derived from the activated partial thromboplastin time, using silica as the activator, are the most frequent techniques. The algorithms for LAC detection are reported here, and every laboratory is encouraged to introduce its own diagnostic procedure. Results should be expressed in ratio to reduce inter- and intravariability. In addition, the effect of anticoagulation in LAC assays and possible strategies for a correct diagnosis are provided.

## 1. Introduction

Lupus anticoagulant (LAC) is a heterogeneous mix of autoimmune antibodies directed against phospholipids (PL) and PL-associated proteins located in cell membranes, such as prothrombin (PT) and β2-glycoprotein I [1]. LAC testing is based on coagulation assays that enhance a phospholipid-dependent inhibitory effect [2]. In this setting, a prolonged activated partial thromboplastin time (APTT) is usually observed.

This interference generates an anticoagulant effect in vitro, although in clinical practice it is associated with a state of hypercoagulability [1]. It was discovered for the first time in 1972 in patients with systemic lupus erythematosus (SLE), although LAC can be detected in other pathologies [3]. An association between LAC and false positive reaction in syphilis test serology (VDRL, Venereal Disease Research Laboratory) has been described [4]. VDRL testing uses an antigen containing cardiolipin, lecithin, and cholesterol, so false positive results can occur.

LAC is one of the laboratory criteria for antiphospholipid syndrome (APS), according to the last classification criteria for APS [5]. Other antiphospholipid antibodies (aPL) are also included in these laboratory criteria: anticardiolipin antibodies and antiβ2-glycoprotein antibodies. All antibodies should be confirmed in two samples separated 12 weeks. The clinical criteria comprise thrombosis (arterial, venous, or small vessel) and/or obstetric morbidity (≥3 consecutive miscarriages before week 10 of gestation, ≥1 miscarriage after week 10, or premature birth before week 34 in the context of eclampsia, preeclampsia, or placental insufficiency).

## 2. Preanalytical Phase

Appropriate preanalytical conditions are essential for a correct LAC diagnosis [6,7]. It is necessary, therefore, to carry out an adequate selection of patients, as well as optimal sample handling.

(1) First of all, to avoid incidental findings that may lead to LAC diagnosis misinterpretation, patients with high APS suspicion must be selected [6]. For this purpose, patients with prolonged unexplained APTT and the rest of the basic hemostasis study within normal parameters (PT time and thrombin time) are candidates for study, especially if thrombosis (arterial or venous) or recurrent miscarriages are associated. It should also be considered in patients < 50 years old who, although they do not meet the clinical criteria, present some autoimmune disorder, such as idiopathic thrombocytopenic purpura, livedo reticularis, or thrombosis, with weak cardiovascular risk factors.
(2) LAC diagnosis should not be performed in the emergency setting unless catastrophic APS (CAPS) is suspected [6]. CAPS is a rare and life-threatening subset of APS characterized by severe thrombotic complications, usually microvascular as well as large vessel, that causes multiorgan failure simultaneously or over a short period of time [7]. In that case, an early diagnosis is required given the severity and mortality associated with this pathology. LAC should not be determined in the acute thrombotic phase or in other pathologies such as infection, surgery, or cancer, in which cytokines are released with the consequent increase in acute phase reactants and factor VIII (FVIII). C-reactive protein has an affinity for the PL present in the reagents, and false positives can occur. Furthermore, an increase in FVIII may shorten the APTT and can induce false negatives. LAC measurement should also be avoided during pregnancy because of the increase in coagulation factors, including FVIII. Anticoagulant treatment can also interfere with the results [6,8].
(3) Regarding the correct handling of the samples, double centrifugation at 2000× *g* for 15 min at room temperature should be carried out in order to obtain platelet-poor plasma (<10 × 10^9^/L) [6,8]. Ultracentrifugation and the use of filters are not recommended, because microparticles are released. In case of freezing, once double centrifugation is performed, the sample should be frozen only once and within the first 4 h after obtaining the blood sample. The sample can be kept frozen for 14 days at −20° and up to 6 months at −70 °C. Hemolyzed, icteric, insufficient, lipemic, or coagulated samples should not be processed [6,8].

## 3. LAC Diagnosis

The heterogeneity of these autoantibodies and the variability of the effect they produce on coagulation tests explains why there is no single test sensitive to all LACs [9]. For this reason, the combination of different techniques is required for a correct diagnosis.

A sequential approach is followed, in agreement with the International Society on Thrombosis and Hemostasis (ISTH) guidelines in 2009 [10], independently of the techniques used:

(1) Screening test: this test uses low PL concentrations. PL of screening tests are heavily di luted to enhance the inhibitory effect of possible LAC, prolonging the APTT beyond the high reference limit. An elongated APTT can be observed not only in the presence of LAC but in other situations such as deficiency of intrinsic pathway factors (FXII, FXI, FIX, FVIII) [6,10].
(2) Mixing test: a mixing of the patient’s plasma and a normal plasma pool (NPP), obtained from at least 20–40 healthy subjects, with factor concentrations around 100%, is performed in a 1:1 proportion without incubation for 30 min. The APTT is determined with a reagent with a low PL concentration. The shortening of APTT indicates a factor deficiency, while if the APTT remains prolonged (>4.5 s compared with the NPP), this suggests the presence of an inhibitor, either LAC (PL-dependent inhibitor) or inhibitors against any intrinsic pathway factor (non-PL-dependent inhibitor). In these situations, clinical data are relevant and can help us orientate the diagnosis. LAC is usually associated with thrombosis, while other inhibitors, for example anti-FVIII in acquired or congenital hemophilia A, are associated with bleeding [6,10].
(3) Confirmatory test: this test uses high PL concentrations provided by the manufacturer. To evidence the PL dependence, the confirmatory test must be performed by increasing the concentration of PL used in the screening test.

If LAC is present, the excess of PL quenches these antibodies, neutralizing LAC. This results in shortening of the APTT in the screening test, which falls into the reference range. In case of an inhibitor different than LAC, the confirmatory test is not affected by elevated PL concentrations, and APTT will be similar to the screening test [6,10].

If clinical suspicion is high, both screening and mixing tests are above the cutoffs, and LAC is not detected by DRVVT (dilute Russell’s viper venom test) or APTT- based techniques (the techniques most frequently performed in the majority of laboratories in hemostasia), using PL such as hexagonal phase phosphatidylethanolamine, purified inosithin, or phosphatidylserine can be a valid approach [10].

In some cases, overall, if a strong LAC is detected, the intrinsic pathway factors can be deficient, because, as has been previously mentioned, LAC is an autoantibody directed against PL and PL-associated proteins located in cell membranes that prolongs PL-dependent clotting times (CTs) [10]. The PL consumption in strong LAC can produce false intrinsic pathway factor deficiencies. This scenario is not usually associated with a bleeding tendency [10].

Because of the heterogeneity of these autoantibodies, at least two techniques with different mechanisms of action must be performed [6,8].

The most commonly used techniques in hemostasis laboratories and following the recommendations of the ISTH are: (1) DRVVT; (2) analysis with a reagent very sensitive to LAC derived from APTT, using silica as the activator (silica clotting time (SCT)) [6,8].

Performing both techniques allows the detection of the vast majority of LAC, with a low incidence of false positives and negatives, and reduces inter- and intraindividual variability. There are different suppliers that provide these techniques, and they may present variability in the PL composition of the reagents and the use of different activators. The lower the concentration of PL and the lower the amount of phosphatidylserine, the higher the sensitivity for LAC detection [1,11]; therefore, it is important to consider the amount and type of PL when choosing the reagent.

DRVVT is considered the test to be performed first because of its specificity and robustness in detecting LAC, as well as its greater correlation with clinical data than with APTT-based techniques. This assay uses a powerful factor X activator, Russell viper venom. The venom contains enzymes that can directly activate not only factor X, but factor V, PT, fibrinogen and plasminogen [1,6,9]. DRVVT can be performed with a screen and confirm reagent pair, the LA1 screening reagent and LA2 confirmatory reagent (Siemens Healthcare, Marburg, Germany). The LA1 reagent is highly sensitive to LAC and initiates clotting by directly activating factor X. The LA2 reagent contains a high PL concentration; the PL excess neutralizes the LAC and corrects the CT [11].

Strong LAC typically prolongs LA1, but weak LAC may not prolong LA1. Such weak LAC can be identified by an abnormal LA1/LA2 ratio. The combination of a prolonged LA1 but normal LA2 indicates the presence of LAC. However, in the rare presence of very strong LAC, the PL content of LA2 may not be sufficient to neutralize all antibodies present in the sample, resulting in a still-prolonged LA2 CT. In this case, performance of LA1 and LA2 on a 1:1 diluted sample in NPP is an option [12].

A prolongation of LA2 suggests a factor deficiency and/or oral anticoagulant therapy with vitamin K antagonists (VKA). In this case, a standard PT time is a simple test to exclude presence of VKA or any other FX, FV, or FII deficiency. If VKA is known to be present, abnormal results for LA1, LA2, and an LA1 mixing study (but a normal result for the mix in LA2) points to the additional presence of LAC, whereas abnormality of all four tests suggests a non-PL-dependent inhibitor [13].

Regarding APTT-based techniques, silica activator has the highest sensitivity for LAC. However, Kumano et al. described an equivalent LAC sensitivity for ellagic acid and silica based APTT reagents with a similar PL concentration [14]. The latest ISTH guidelines recommend the use of an APTT reagent with a low PL concentration and silica as the activator of choice, but it has also been reported that ellagic acid has an acceptable sensitivity, at least in some APTT reagents, and every laboratory should assess the sensitivity of its APTT reagents [1,6,9].

There are other assays for studying LAC, but they are not widely used because of unfamiliarity, variable availability, poor reproducibility, and technical issues [15,16].

- Dilute prothrombin time (dPT, extrinsic pathway-based test): the high PL concentration in thromboplastin reagents for PT time is such that LAC rarely prolongs routine PT time [6]. Dilution of the thromboplastin to generate a dPT screening test increases LAC sensitivity. A lower thromboplastin dilution, or even undiluted reagent, can be used as the dPT confirmatory test. The dPT assay triggers coagulation via tissue factor and factor VII and contains heparin neutralizer. Although reproducibility is improved because of minimal variation inter- and intralaboratories, it is not available in the majority of laboratories [15,16].
- ASLA assay (activated seven lupus anticoagulant assay, extrinsic pathway-based test): coagulation is initiated by recombinant factor VII, independently of tissue factor [6]. Dilute and concentrated PL are used for screening and confirmatory tests. This assay is not commercialized [6,15,16].
- Kaolin clotting time (KCT, intrinsic pathway-based test): this test uses kaolin as the contact activator. PL are very dilute, making it sensitive to LAC [12,13]. However, KCT is not compatible with some optical analyzers and has poor reproducibility. KCT does not have any commercial confirmatory test, reducing specificity of KCT for LAC [6,15,16].
- Vipera lebetina venom time (VLVT, common pathway-based tests): this assay is similar to DRVVT. Macrovipera lebetina venom activates factor X, and low and high PL screen and confirm reagents are available. Equivalence with DRVVT has not been proven, but it is commercially available. Reagents contain heparin neutralizer [6,15,16].
- Textarin and ecarin time (common pathway-based tests): these assays are provided with snake venom’s factor II activators (Pseudonaja textilis and Echis Carinatus, respectively). They activate the undercarboxylated factor II form produced on VKA, converting it to thrombin or meizothrombin, bypassing factor Xa, and are, therefore, an adequate option in anticoagulated patients. Textarin is a portion of Pseudonaja textilis venom sensitive to PL, factor V, and calcium. Therefore, diluting PL makes textarin sensitive to LAC [6,12,13]. However, the ecarin fraction is cofactor independent, so the lack of PL does not prolong CT if LAC is present. This property makes ecarin time an optimal confirmatory test [6,15,16].
- Taipan snake venom time (TSVT, common pathway-based tests): TSVT is a dilute PL screening test based on factor II activation via Oscutarin C fraction of Oxyuranus scutellatus venom [6]. Pairing TSVT with ecarin time has shown to improve LAC detection in anticoagulated patients. In fact, Moore et at designed an international multicenter study, validating the TSVT and ecarin time pairing for LAC measurement in nonanticoagulated patients and those on VKA and with direct factor Xa inhibitors [17].

**Figure 1:**
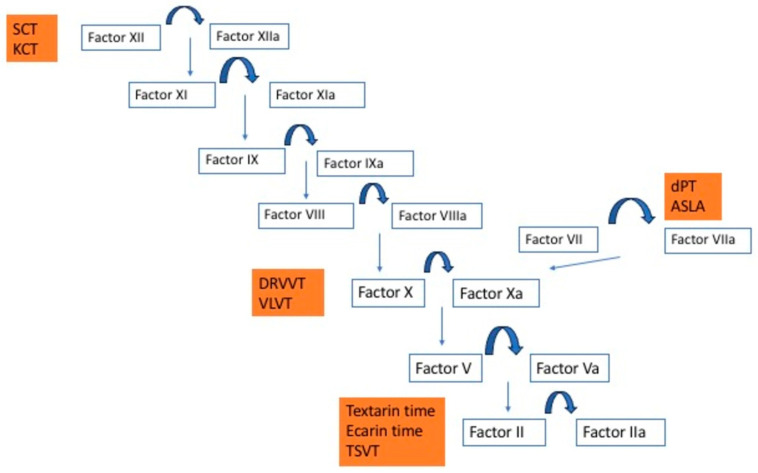
LAC techniques and activation of the coagulation cascade. Abbreviations: SCT: silica clotting time; KCT: kaolin clotting time; dPT: dilute prothrombin time; ASLA: activated seven lupus anticoagulant; DRVVT: dilute Russell viper venom time; VLVT: vipera lebetina venom time; TSVT: Taipan snake venom time.

See Figure 1 for the coagulation cascade activation by each LAC technique.

## 4. Interpretation of Results

The screening, mixing, and confirmatory CT are susceptible to inter- and intraindividual variability, so normalizing the CT by generating a ratio between the patient’s CT and the NPP is highly recommended.

(1) Screening tests suggest LAC or intrinsic pathway factor deficiency if the ratio is above the cutoff [1,8].
(2) Mixing tests: There are two ways to interpret mixing time results, the use of circulant anticoagulation index (ICA or also called Roxner index) and the mixing-test-specific cutoff point (MTC). The ICA results from the following formula: ([1:1 mix Sample (s) − NPP (s)]/screen Patient (s)) × 100 [1].

An ICA result > 12% suggests the presence of LAC. However, the ICA has been shown to be less sensitive to LAC than the MTC, expressed as a normalized ratio. The ISTH, therefore, recommends the MTC and not the ICA [8]. The cutoff can be established in each hemostasis laboratory (homemade), and ideally standardized criteria should be used for statistical analysis to determine the ideal 99th percentile in 120 healthy volunteers. This is not usually affordable, since this procedure requires a high number of healthy subjects, it is time-consuming and expensive, and variability in reagents and between laboratories has been described. This cutoff can also be provided by the supplier; it is normally verified in 20–40 healthy subjects, and it is assumed that an adequate statistical study has been established for its determination [6,18].

(3) Confirmatory: The percentage for CT correction after adding PL results from the following formula: [(screening CT − confirmatory CT)/screening CT] × 100. If the percentage of correction is above the cutoff, this suggests LAC [1,8].

The final interpretation is positive or negative for LAC detection. Each hemostasis laboratory must implement its own diagnostic algorithm. LAC is detected (positive result) if the ratio APTT (screening test)/APTT (confirmatory test) > 1.30, doubtful if it is between 1.20 and 1.30, and negative if it is below 1.20 [1,8].

## 5. Algorithms for LAC Detection

**Figure 2:**
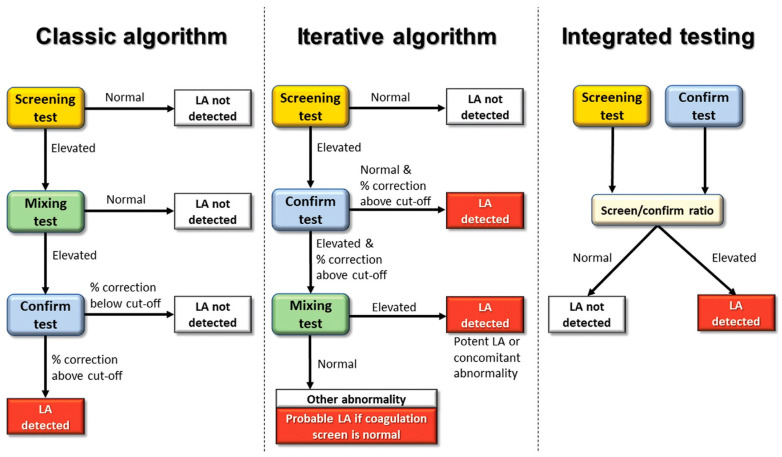
LAC detection algorithms. Reprinted with permission from reference [6]. Copyright 2022 Georg ThiemeVerlag KG.

Three algorithms for LAC detection have been described. See Figure 2.

(1) Classic algorithm: this algorithm generates a sequential exclusion format that allows LAC to be distinguished from other causes of prolonged APTT. It has been criticized for the presence of false negatives in the mixing test and the nondetection of weak positive LAC [6]. It should be noted that not all NPPs are identical and that CT may vary, such that an NPP with a shorter APTT requires a more powerful LAC to increase the APTT above the reference value [19].

(2) Iterative algorithm: this algorithm assumes that a screening with a prolonged APTT without a justified cause corrected with the confirmatory test is sufficient to detect LAC and omits the mixing test [6]. In cases of the coexistence of LAC and intrinsic pathway factor deficiency, the screening and confirmatory tests show prolonged APTT. The mixing test can be useful in these situations, since it corrects the factor deficiency and dilutes the antibody. This discrepancy between screening and confirmatory testing with a correcting mixing test may lead to this coexistence [6].

(3) Integrated algorithm: in this algorithm, screening and confirmatory testing are performed in parallel, and the mixing test is included for uncertain cases [6]. It has the advantage of detecting LAC in a single step and identifying weak LAC, in which the prolongation of APTT is insufficient to exceed the normal cutoff but the discrepancy between screening and confirmatory testing suggests LAC. However, this algorithm may not be valid for every patient, as it simplifies the detection of LAC [20].

The Clinical and Laboratory Standards Institute (CLSI) has established the screening–confirmatory–mixing order [21]. The mixing test is performed if the following criteria are met: (1) no evidence of other causes of prolonged coagulation times; (2) screening with prolonged APTT; (3) APTT correction with the confirmatory test.

The ISTH and the British Society of Hematology (BSH) recommend performing the confirmatory and the mixing tests if the APTT in screening is prolonged (iterative algorithm) [6,22].

An automatic algorithm integrated into the STA-Coag Expert software (Stago, Asnières, France), based on the ISTH recommendations, was performed by Florin et al. [23]. The ICA has been used for the mixing test. The authors analyzed 194 samples and found a sensitivity of 94% and a specificity of 100% for LAC detection. There were 3 false negatives and 11 doubtful mixtures with negative confirmatory results. The time to obtain a result was reduced from 200 to 80 min. This automatic algorithm was comparable to manual LAC interpretation and may be useful in the hemostasis laboratories.

## 6. Effect of Anticoagulants on LAC Assays

(1) Heparins.

Heparins can prolong screening, mixing, and confirmatory CT, so they can make correct LAC interpretation difficult [1].

The ISTH recommendations to minimize interferences are [11,24]: (a) measuring the anti-Xa activity of low molecular weight heparins—if anti-Xa activity is within the therapeutic range, neutralizers (heparinases or polybrene) can be used; (b) measuring the thrombin time, which indicates the presence of heparin and is unsensitive to LAC; (c) determining LAC at the heparin trough level (just before the next heparin administration).

(2) Vitamin K antagonists (VKA).

All LAC detection techniques can be affected by VKAs except those that use PT activators derived from snake venoms (limited availability) [1].

The 2009 ISTH guidelines [10], CLSI [21], and BCH [22] consider the option of performing a 1:1 mixture with normal plasma, which can correct the factor deficiency induced by VKAs, especially if INR is between 1.5 and 3. However, the current ISTH guidelines [6] point out that this method may not be reliable, since the degree of correction depends on the reagent used and there may be false negatives due to the LAC activity possibly being diluted with the mixture.

Possible alternatives are: (a) a temporary change to low-molecular-weight heparin, with prior determination of its administration (trough level, no heparin effect detected); (b) the use of PT activators derived from snake venoms, which have good sensitivity and specificity in anticoagulated patients, although they are not available in routine laboratories.

(3) Direct oral anticoagulants (DOACs)

All LAC detection techniques can be affected by DOACs except those that use PT activators derived from snake venoms (limited availability) [1,24]. All DOACs prolong DVVRT in screening and confirmatory testing. Rivaroxaban prolongs screening to a greater extent (increases false positives), while apixaban prolongs confirmatory testing (increases false negatives).

The ISTH recommendations [8] for a correct determination of ACL in patients taking DOACs are:

(a) Discontinue the DOAC and adjust according to renal function, as established by the DOAC withdrawal guidelines before surgery or a scheduled invasive procedure. The worse the kidney function, the more days of DOAC withdrawal will be necessary to ensure that there is no DOAC activity. The anti-Xa activity of rivaroxaban, apixaban, and edoxaban and the anti-IIa activity of dabigatran could be measured, if available, in the hemostasis laboratory, and LAC study could be carried out if their effect is not detected.
(b) Use neutralizers. There are two commercialized DOAC removing agents: DOAC-stop^®^ (Haematex Research, Hornsby, Australia) and DOAC-remove^®^ (5 Diagnostics, Basel, Switzerland). These neutralizers contain active charcoal compounds able to eliminate DOAC molecules in plasma. Briefly, absorbents and patient plasma are mixed, and then a short incubation and centrifugation of this mixture is performed [1,25]. The supernatant plasma, which is supposed to be DOAC-free, is used for LAC measurement. In addition, filtration techniques can also remove DOAC from the patient plasma [26]. All these neutralizers do not guarantee complete DOAC removal, and an effect on CT may be detected. Therefore, false positives and negatives have been described [1]. Wang et al. studied the impact of DOAC-stop^®^ on thrombin generation assays in NPP before and after adding different anti-Xa DOAC concentrations [27]. In this study, the majority of DOAC levels were <1 ng/mL, except for that of one sample spiked with 502 ng/mL of apixaban, which was reduced to 6 ng/mL. Regarding thrombin generation parameters, a significant increase in the peak height (21.9%) and velocity index (42.6%) were detected following the addition of DOAC-stop^®^. The decrease in tissue factor pathway inhibitor (TFPI) could explain these findings. Strong correlations were described before and after DOAC-stop^®^ addition for all parameters [27]. They concluded that global assays can be an option for studying the thrombotic state in these patients.
(c) Use of PT activators, not applicable if the patient takes dabigatran.

Pengo and collaborators [28] propose: (1) ask about personal and family history of autoimmunity; (2) determine aPL—if negative, start DOACs, and if positive, determine PT/PS (phosphatidylserine) antibodies. If they are positive, LAC is usually positive (tetrapositivity).

## 7. Other Specific Clinical Issues

Small vessel thrombosis is part of the clinical manifestations of APS, and renal, hepatic, retinal, skin, and pulmonary thrombosis can occur, among other sites of the microvasculature [1]. Renal microvascular lesions have long-term poor outcomes, in particular if associated with thrombotic microangiopathy (TMA), described in 25% of patients with lupus nephritis [29]. These patients had more severe clinicopathological features than patients without coexistent TMA, with higher rates of oliguria, more advanced kidney injury, and more extensive fibrocellular/fibrous crescents and tubular atrophy. Moreover, their prognosis was worse, with lower rates of clinical remission and higher rates of treatment failure and death [29].

APS is related to TMA (primary or secondary to SLE and other autoimmune diseases), thrombotic thrombocytopenic purpura (TTP), hemolytic uremic syndrome, HELLP (hemolysis, elevated liver enzyme levels, and low platelet levels) syndrome, and/or CAPS [30]. Although a severe ADAMTS13 (a disintegrin and metalloproteinase with a thrombospondin type 1 motif, member 13) activity (<10%) is a characteristic finding of TTP, its reduced activity has also been documented in other pathologies, including SLE, scleroderma, and APS [31]. A subset of APS has been proposed apart from the classic and CAPS [30,31,32]. Other papers have described the concurrence of APS and TTP [33,34]. Espinosa et al. showed that in 46 cases of TMA hemolytic anemia with aPL, APS was diagnosed in 61% of them [35]. The role of aPL in the microangiopathy is not clear. An endothelial cell activation with upregulation of tissue factor expression has been proposed to explain the proinflammatory and prothrombotic properties of aPL. In addition, aPL facilitate platelet aggregation and complement cascade activation. As a consequence, the complement system damages the vascular endothelium, enhancing the procoagulant state and thrombosis development [32]. In acquired TMA, the reduction in ADAMTS13 activity is mainly due to the presence of antibodies against ADAMTS13, but other mechanisms are implied, such as its inhibition by inflammatory cytokine interleukin (IL-6) or cleavage by protease released from neutrophils [32].

Lee et al. indicated that a reduction in ADAMTS13 activity is a significant thrombotic risk factor in patients with aPL, independently of the presence of anti-ADAMTS13 [31]. These authors included 216 patients (88 men and 128 women), 40 (18.5%) with thrombosis. Eighty-seven patients had a reduced ADAMTS13 activity, twenty-five (62.5%) among the patients with thrombosis and sixty-two (35.2%) in the nonthrombotic group (*p* = 0.013). Severe ADAMT13 activity (<10%) was described in 11 patients, 2 with thrombosis (5% of thrombotic patients). However, anti-ADAMTS13 did not significantly increase the risk of thrombosis. In patients with reduced ADAMTS13 activity and aPL, ADAMTS13 activity should be monitored, and the clinician has to check for thrombocytopenia and hemolytic anemia, the presence of schistozytes, and other clinical and laboratory parameters to discard TMA. The clinician should also initiate exchange plasma and corticosteroids if needed.

LAC may occasionally be associated with PT deficiency [36]. The cause of this deficiency is the presence of a high-affinity, non-neutralizing anti-PT antibody, which upon binding to prothrombin in vivo creates an immune complex that is cleared from the circulation by the monocytic–macrophage system. This condition is known as LAC-associated hypothrombinemia. It is important to identify these patients, because critical hemorrhagic complications can appear [36].

Patients with LAC and/or other aPL without thrombosis should be followed in the outpatient clinic of hemostasia. Optimal control of the main cardiovascular risk factors is important to minimize thrombotic risk, and antiaggregation in high-risk patients should be considered. If the patient develops thrombosis, anticoagulation is required, preferably with VKA. DOACS can be considered in low-risk aPL profiles or in cases of intolerance or failure to VKA [37]. Long-term anticoagulation is necessary because of the high recurrence rate. A recurrence rate of 11% even with high-intensity anticoagulation has been described [38].

## 8. Conclusions

The heterogeneity of LAC implies a frequent challenge in the hemostasis laboratory, so there is no test that alone detects all LAC. Two techniques with different mechanisms of action must be combined: (1) DRVVT; (2) analysis with a very sensitive reagent to LAC derived from APTT, using silica as activator.

A correct preanalytical phase is essential to minimize interference for a correct LAC diagnosis. Screening, mixing, and confirmatory CT scans are susceptible to inter- and intraindividual variability. Therefore, it is recommended to normalize the CT scan by generating a ratio between the patient’s CT and the NPP CT. The ISTH and BSH recommend performing the confirmatory and mixing tests if the APTT in screening is prolonged (iterative algorithm). The CLSI suggests the screening–confirmatory–mixing order, with the mixing test to be performed if APTT corrects with the confirmatory test. However, each hemostasis laboratory must implement its own diagnostic algorithm based on its own criteria and resources. The final interpretation is LAC positive/negative. LAC detection techniques may be altered by taking anticoagulants. APS, TMA, and LAC-associated hypothrombinemia are particular clinical situations that require early diagnosis because of their high morbidity and mortality.

## Abbreviations

The following abbreviations are used in this manuscript:

ADAMTS13	A disintegrin and metalloproteinase with a thrombospondin type 1 motif, member 13
aPL	Phospholipid antibodies
APS	Antiphospholipid syndrome
APTT	Activated partial thromboplastin time
ASLA	Activated seven lupus anticoagulant assay
BSH	British Society of Hematology
CAPS	Catastrophic antiphospholipid syndrome
CLSI	Clinical and Laboratory Standards Institute
CT	Clotting times
DOAC	Direct oral anticoagulants
DPT	Dilute prothrombin time
DRVVT	Dilute Russell’s viper venom test
HELLP	Hemolysis, elevated liver enzyme levels, and low platelet levels
ICA	Index of circulating anticoagulant
INR	International normalized ratio
ISTH	International Society on Thrombosis and Hemostasis
KCT	Kaolin clotting time
LAC	Lupus anticoagulant
MTC	Mixing-test-specific cutoff
NPP	Normal plasma pool
PL	Phospholipids
PT	Prothrombin
PS	Phosphatidylserine
SCT	Silica clotting time
SLE	Systemic lupus eritematosus
TFPI	Tissue factor pathway inhibitor
TMA	Thrombotic microangiopathy
TSVT	Taipan snake venom time
TTP	Thrombotic thrombocytopenia purpura
VDRL	Venereal Disease Research Laboratory
VKA	Vitamin K antagonists
VLVT	Vipera lebetina venom time
